# Immunoregulation by Artemisinin and Its Derivatives: A New Role for Old Antimalarial Drugs

**DOI:** 10.3389/fimmu.2021.751772

**Published:** 2021-09-09

**Authors:** Feifei Qiu, Junfeng Liu, Xiumei Mo, Huazhen Liu, Yuchao Chen, Zhenhua Dai

**Affiliations:** ^1^Section of Immunology & Joint Immunology Program, Guangdong Provincial Academy of Chinese Medical Sciences & Guangdong Provincial Hospital of Chinese Medicine, Guangzhou, China; ^2^State Key Laboratory of Dampness Syndrome of Chinese Medicine, the Second Affiliated Hospital of Guangzhou University of Chinese Medicine, Guangzhou, China; ^3^Guangdong Provincial Key Laboratory of Clinical Research on Traditional Chinese Medicine Syndrome, The Second Affiliated Hospital of Guangzhou University of Chinese Medicine, Guangzhou, China; ^4^Guangdong-Hong Kong-Macau Joint Lab on Chinese Medicine and Immune Diseases, Guangzhou University of Chinese Medicine, Guangzhou, China

**Keywords:** artemisinin, adaptive immunity, innate immunity, autoimmune disease, immunoregulation, cellular signaling

## Abstract

Artemisinin and its derivatives (ARTs) are known as conventional antimalarial drugs with clinical safety and efficacy. Youyou Tu was awarded a Nobel Prize in Physiology and Medicine due to her discovery of artemisinin and its therapeutic effects on malaria. Apart from antimalarial effects, mounting evidence has demonstrated that ARTs exert therapeutic effects on inflammation and autoimmune disorders because of their anti-inflammatory and immunoregulatory properties. In this aspect, tremendous progress has been made during the past five to seven years. Therefore, the present review summarizes recent studies that have explored the anti-inflammatory and immunomodulatory effects of ARTs on autoimmune diseases and transplant rejection. In this review, we also discuss the cellular and molecular mechanisms underlying the immunomodulatory effects of ARTs. Recent preclinical studies will help lay the groundwork for clinical trials using ARTs to treat various immune-based disorders, especially autoimmune diseases.

## Introduction

Artemisinin, originally extracted from Artemisia annua or Qinghao, is a safe and effective drug for the treatment of malaria (1). In 1972, artemisinin was first discovered by Youyou Tu (2) who was then awarded a Nobel Prize in Physiology and Medicine in 2015. The chemical structure of artemisinin was determined to be a sesquiterpene endoperoxide, which is totally different from that of other conventional antimalarial drugs (3). Subsequently, some artemisinin-based derivatives with better bioactivity or solubility have been synthesized, including dihydroartemisinin, artesunate, artemether, SM934, DC32 and ADART (9,10-Anhydrodehydroartemisin) (4). Later, artemisinin-based combination therapies were recommended by the World Health Organization (WHO) for the treatment of malaria, although artemisinin and its derivatives (ARTs) were considered as the first-line antimalarial drugs. Long known for their antimalarial effects, ARTs have recently exhibited other pharmacological properties, such as antitumor (5), antiviral (6), anti-fibrotic (7, 8) and anti-inflammatory effects (9, 10). The mechanisms underlying the effects of ARTs on inflammation have been briefly described in a review by An et al., with a focus on the impacts of quinoline- and acridine-based antimalarial drugs on innate immunity and autoimmune diseases (9). Meanwhile, immunosuppressive features of ARTs have also been briefly reviewed based on earlier studies on autoimmune diseases (10). However, in terms of immunomodulatory effects of ARTs, tremendous progress has been made during the last five to six years. We have recently demonstrated that dihydroartemisinin, an artemisinin derivative, ameliorates psoriatic skin inflammation and its relapse by selectively diminishing memory CD8+ T cells (11). In this review, we summarize recent studies that have explored the anti-inflammatory and immunomodulatory effects of ARTs on autoimmune diseases and allograft rejection as well as the mechanisms underlying their actions. We also briefly discuss the potential application of ARTs in clinic.

## Artemisinin Family Drugs Exert Immunoregulatory Effects on Immune-Mediated Inflammation or Autoimmune Diseases as Well as Allograft Rejection

**Figure 1:**
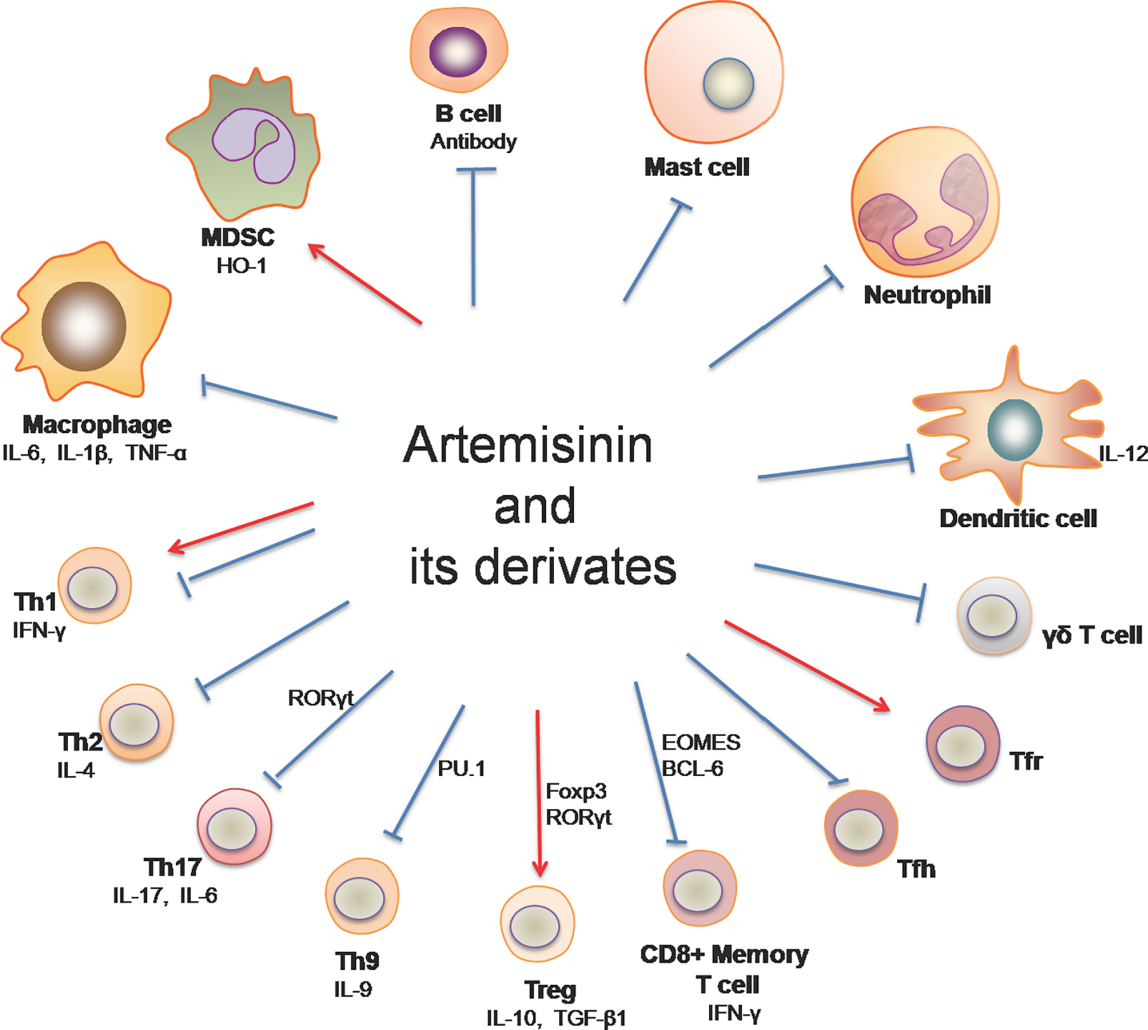
Artemisinin and its derivatives on both adaptive and innate immune cells. Artemisinin and its derivatives have the capacity to regulate expressions of proinflammatory and anti-inflammatory cytokines, the frequency and activation of T helper and B cells, and the responsiveness of macrophages, DCs, neutrophils, mast cells and MDSCs. “↓“ denotes “enhancing” while “⊥“ indicates “suppressing”. (Th1, T helper 1 cell; Th2, T helper 2 cell; Th9, T helper 9 cell; Th17, T helper 17 cell; Treg, regulatory T cells; Tfh, follicular helper T cells; Tfr, follicular regulatory T cells; MDSC, myeloid-derived suppressor cells).

Immune-mediated inflammatory diseases (IMIDs) are a group of common and chronic disorders characterized by dysregulation of the immune system, resulting in inflammation and damage to target organs (12, 13). Examples of IMIDs include inflammatory bowel disease (IBD), psoriasis, rheumatoid arthritis, multiple sclerosis and systemic lupus erythematosus (SLE) (14), most of which are known to be autoimmune diseases. On the other hand, allograft rejection mediated by innate and adaptive immunity remains a main cause of graft failure after transplantation, posing a challenge to transplant patients’ quality of life and survival. Artemisinin and its derivatives (ARTs) have been shown to impact immune cells (**Figure 1**) and exert therapeutic effects on allograft rejection and IMIDs, including rheumatoid arthritis, psoriatic skin inflammation, IBD, multiple sclerosis, SLE and IgA nephropathy.

## Rheumatoid Arthritis

RA is a chronic inflammatory disease with the feature of Treg/Th17 imbalance, resulting in cartilage degradation and bone erosion within both small and large joints (15). The exact cause of RA remains unknown, posing a challenge to the diagnosis and treatment for RA (16). Accumulating evidence based on animal models has pointed to the efficacy of ARTs in RA treatment. In type II collagen-induced arthritis (CIA) in rats, artesunate treatment not only alleviated inflammation, decreased the frequency of Th17 cells and increased Treg cells in the synovium and spleen (17), but also stimulated apoptosis or autophagy in cartilage tissue and inhibited chondrocyte proliferation through the PI3K/AKT/mTOR signaling pathway (18). Moreover, artesunate exerted a suppressive effect on osteoclastogenesis and improved arthritic bone erosion in CIA rats via inhibiting the production of ROS and activating antioxidant enzyme as well as p62/Nrf2 signaling (19). In addition, artesunate significantly inhibited the migration and invasion of fibroblast-like synoviocytes (FLS) in patients with RA by suppressing PDK1-induced activation of AKT and RSK2 phosphorylation as well as MMP-2 and MMP-9 production (20). Similarly, DC32 [(9α,12α-dihydroartemisinyl)bis(2’-chlorocinnmate)], a dihydroartemisinin derivative, remarkably dampened footpad inflammation, reduced cartilage degradation through the Nrf2-p62-Keap1 feedback loop in DBA/1 mice with CIA (21), and impeded cellular infiltration and inflammation *via* restoration of Treg/Th17 balance and downregulation of the expression of IL-6, resulting in ultimate attenuation of RA (22). These data have suggested that ATRs can effectively alleviate RA by regulating Th17/Treg balance, FLS mobility and oxidative stress *via* suppressing PI3K/AKT/mTOR and AKT/RSK2 signaling pathways while activating p62/Nrf2 signaling.

## Immune-Mediated Skin Diseases: Psoriasis and Atopic Dermatitis

Psoriasis, which affects over 60 million people worldwide, is an inflammatory or autoimmune skin disease (23). An important role for T cells in the pathogenesis of psoriasis was confirmed by several clinical trials (24, 25), while tissue-resident memory T cells (T_RM_) reportedly led to the recurrence of psoriasis at sites of previously involved skin (26). We recently found that dihydroartemisinin is more effective than methotrexate in suppressing psoriasis relapse. Dihydroartemisinin not only reduced acute skin lesions and recurrence of psoriasis in imiquimod (IMQ)-induced psoriasis-like mice, but also ameliorated psoriatic human skin lesions in humanized NSG mice receiving lesional skin from patients with psoriasis (11), mainly by diminishing CD8+ central memory T (T_CM_) and resident memory T (T_RM_) cells (11). Furthermore, artesunate was also reported to inhibit epidermal thickening and systemic inflammation in IMQ-induced psoriatic mice by reducing γδ T cells in the draining lymph nodes (27).

Atopic dermatitis (AD) is a common and chronic inflammatory skin disease with severe pruritus, cutaneous and systemic immune dysfunction, and skin lesions. Although the exact pathogenesis of AD remains unclear, it likely results from interactions of genetic and environmental factors (28, 29). It was reported that dihydroartemisinin could ameliorate AD symptoms and skin lesions in DNCB-induced AD mouse models. At high doses, dihydroartemisinin significantly alleviated mast cell infiltration into the skin lesions (30), indicating that it exerts therapeutic effects on AD by targeting mast cells in the skin. Similarly, artesunate relieved AD symptoms and mast cell infiltration, mainly by decreasing the expression of proinflammatory cytokines, including IL-6, IL-17 and IL-23, and suppressing RORγt and STAT3 phosphorylation (31). Taken together, these studies have indicated that ARTs attenuate both psoriasis and AD through inhibition of CD8+ memory T cells, Th17/γδ T cells and mast cells.

## Inflammatory Bowel Disease

IBD is a chronic inflammatory bowel disorder mediated by autoimmunity, leading to the injury of gastrointestinal tracts (32, 33). IBD mainly includes ulcerative colitis (UC) and Crohn’s disease (34). In a dextran sulfate sodium (DSS)-induced mouse colitis model, artemisinin was shown to downregulate LYVE-1+ lymphatic vessel density and ameliorate the intestinal inflammation by inhibiting VEGF-C/VEGFR-3-mediated lymphangiogenesis and infiltration of macrophages and neutrophils in colon tissue (35), promoting macrophage polarization toward an M2 phenotype and/or suppressing the process of epithelial-mesenchymal transition (36). Besides, dihydroartemisinin and artesunate have been reported to effectively alleviate colitis symptoms in mice *via* different cellular and molecular mechanisms. For example, treatment with dihydroartemisinin suppressed activation of PI3K/AKT and NF-*κ*B signaling pathways (37), promoted CD4+ T cell apoptosis and restored Th1/Treg cell balance through enhancing heme oxygenase-1 (HO-1) production (38). Dihydroartemisinin also regulated the expression of proinflammatory genes and cell junction-associated genes and normalized the abundance of the gut bacteria that was altered in colitis mice (39). Furthermore, artesunate reportedly reduced expression of IFN-γ, IL-17, and TNF-α in experimental colitis (40), inhibited TLR4-NF-κB signaling pathway (41), promoted apoptosis of macrophages and DCs, and reduced TNF-α and IL-12 production *in vivo* and *in vitro (* 42) while suppressing excessive ER stress (43), cell apoptosis and inflammatory responses *via* the NF-κB pathway (44, 45). Thus, ARTs exert immunoregulatory effects on various immune cells, including T helper cells, Tregs, macrophages, neutrophils and DCs, by modulating NF-κB and PI3K/AKT signaling pathways, resulting in an improvement of colitis symptoms.

## Multiple Sclerosis

MS is a chronic immune-mediated disease of the central nervous system, resulting in the destruction of oligodendrocytes and myelin sheaths and impairment of mobility and cognitive processing (46). Although MS etiology is complex and not completely elucidated, it seems to result from a combination of environmental, genetic and epigenetic factors (47). The therapeutic efficacy of artemisinin family drugs on MS was investigated using a mouse model of experimental autoimmune encephalomyelitis (EAE). It was found that artemisinin ameliorated EAE and reduced plaque formation in the brain with a decrease in IFN-γ expression and an increase in IL-4 production (48). Administration of artesunate attenuated the clinical signs and symptoms of EAE *via* preventing migration of pathogenic T cells to the central nervous system (49). Furthermore, Lv found that 9,10-Anhydrodehydroartemisin (ADART), a compound derived from artemisinin, effectively reduced inflammation in the central nervous system by inhibiting Th1 and Th17 cells (50). Therefore, the therapeutic effects of ARTs on MS may be attributed to their suppression of Th1 and Th17 cells.

## Systemic Lupus Erythematosus

As a chronic and systemic autoimmune disease, SLE is characterized by the dysfunction of immune cells, the production of a wide range of autoantibodies and the formation of immune complexes (51, 52). It was reported that dihydroartemisinin suppressed LPS-induced activation and proliferation of spleen cells from lupus-prone MRL/lpr mice possibly through inhibiting TLR4 expression and IRF3 phosphorylation (53). Li et al. showed that senescence of myeloid-derived suppressor cells (MDSCs) promoted the pathogenesis of SLE, while dihydroartemisinin alleviated the manifestation of SLE by attenuating MDSC senescence *via* regulating Nrf2/HO-1 pathway (54). Subsequently, it was demonstrated that dihydroartemisinin alone, or in combination with prednisone treatment, significantly ameliorated the signs and symptoms of murine SLE through restoring the Treg/Th17 balance by reducing transcription of RORγt and increasing expression of Foxp3 in T cells (55). Serum levels of Macrophage migration inhibitory factor (MIF) in SLE patients were positively associated with the disease activity. Artesunate was shown to decrease MIF level in HUVEC culture with IFNα stimulation and in SLE patient-derived PBMC culture, partly through attenuating STAT1 phosphorylation, indicating a potential therapeutic effect of artesunate on SLE-associated atherosclerosis (56). Another study *in vivo* revealed that artesunate ameliorated the symptoms of lupus nephritis, decreased renal deposition of anti-dsDNA antibodies and suppressed the production of pathogenic cytokines through a reduction of follicular T helper cells (Tfh) and enhancement of follicular regulatory T cells (Tfr) as well as suppression of Jak2-Stat3 signaling pathway (57). Besides, SM934, an artemisinin derivative, extended the lifespan of MRL/lpr mice, relieved the lymphadenopathy symptoms, and suppressed B cell activation and plasma cell formation *in vivo (* 58). Thus, ARTs appear to exert therapeutic effects on SLE, and the mechanisms underlying their effects were likely dependent on their regulation of MDSC senescence, Treg/Th17 balance and/or Tfh/Tfr ratio through Nfr2/HO-1 and Jak/STAT signaling pathways.

## IgA Nephropathy

IgA nephropathy (IgAN) is a common glomerular disease and one of the leading causes of end-stage renal diseases (59). IgAN is an autoimmune disease characterized by IgA immunodeposits within the mesangium of the glomeruli, usually resulting in hematuria and renal functional insufficiency (60). An animal study performed by Bai and his colleagues demonstrated that in combination with hydroxychloroquine (AH), artemisinin significantly improved renal function, decreased IgA and IgG depositions, and reduced the expression of nuclear NF-κB and NLRP3 inflammasome-related proteins while elevating the secretion of exosomes in the kidney. They revealed that increased exosomes from HK-2 cells could inhibit the NF-κB signal pathway and NLRP3 inflammasome activation (61). On the other hand, the authors also found that artemisinin in combination with hydroxychloroquine ameliorated IgAN by reducing Th2 and Th17 cells while increasing Treg cells (62). Subsequently, it was also shown that dihydroartemisinin downregulated the mTOR/S6K1 signaling pathway in human mesangial cells (HMCs), promoted cell autophagy and suppressed cell proliferation in IgA1-stimulated HMCs, suggesting that dihydroartemisinin is a novel mTOR inhibitor and can exert an anti-proliferative effect on HMCs in IgAN (63).

## Transplant Rejection

Allograft rejection represents an immune process in which the donor alloantigens evoke a vigorous immune response of a recipient against a transplant, contributing to graft loss (64). Therefore, more effective therapies need to be explored to improve transplant outcomes as current immunosuppressive agents may cause various side effects. An important study by Yang et al. revealed that artemisinin alleviated not only T cell-mediated but also antibody-mediated rejection in a cardiac transplant rat model by regulating the balance of T effector and Treg (Teff/Treg), impeding B cell activation and antibody production, and decreasing macrophage infiltration in an allograft, resulting in prolongation of graft survival. Moreover, they reported that artemisinin inhibited the activation or function of T cells, B cells and macrophages *in vitro (* 65). Another study indicated that artemisinin remarkably extended survival time of murine skin allografts without significant changes of CD4^+^CD44^hi^CD62L^hi^ T cells *in vivo*. However, artemisinin reduced OX40^+^ T cell frequency and IL-6 secretion *in vitro* (66). Thus, artemisinin can exert immunosuppressive effects on alloimmunity or allograft rejection and may be implicated in clinical transplantation.

## The Effects of Artemisinin and Its Derivatives on Cellular Signaling Pathways

### NF-κB

**Figure 2:**
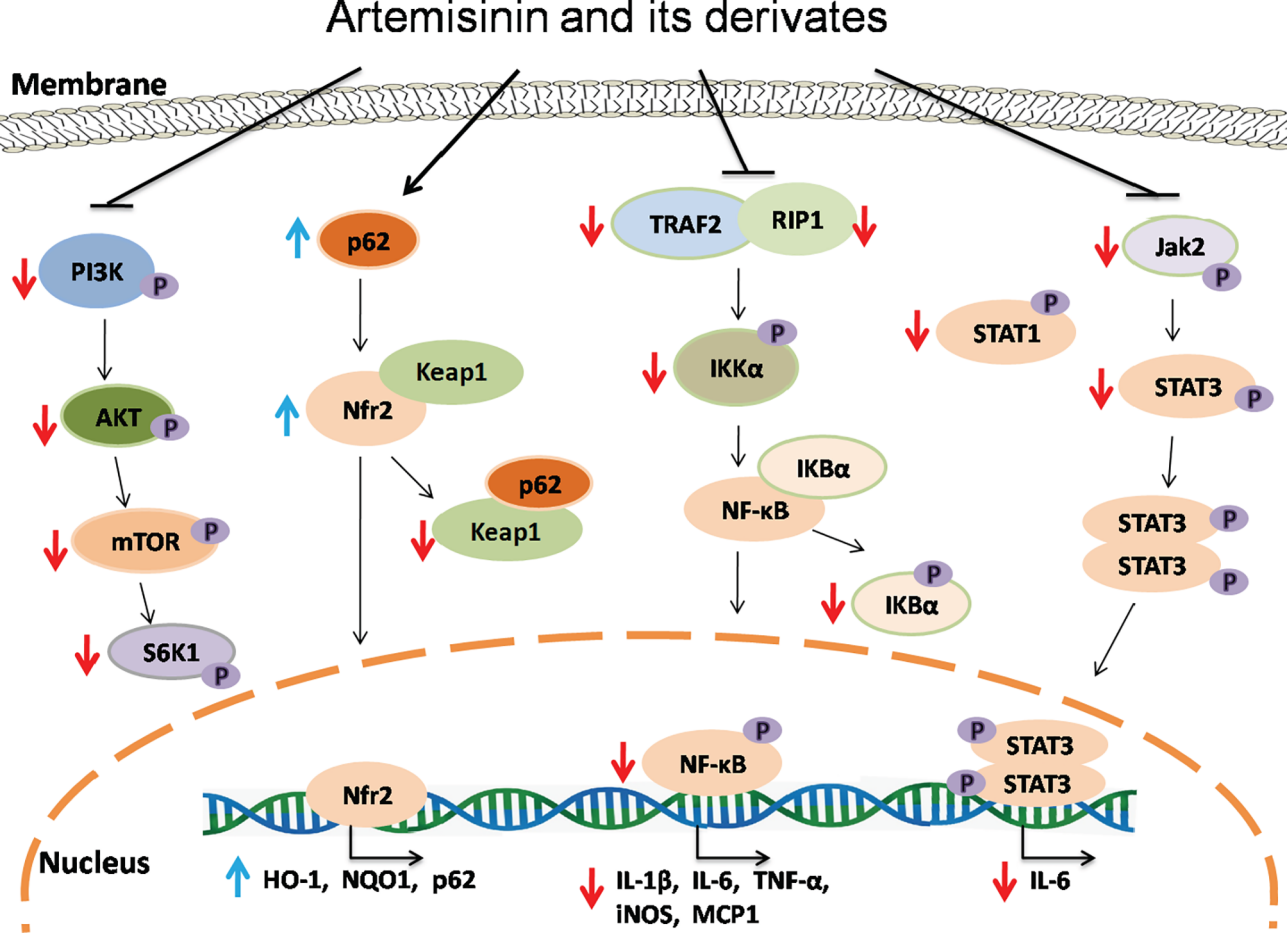
The signaling mechanisms underlying effects of artemisinin and its derivatives. Artemisinin and its derivatives exert immunoregulatory and anti-inflammatory effects *via* interfering with NF-kB, Nrf2, Jak/STAT and mTOR signaling pathways, resulting in downregulation of proinflammatory genes and upregulation of anti-inflammatory and antioxidant genes. Red downward arrows indicate suppressive effects, while blue upward arrows denote stimulating effects.

The nuclear factor-kappa B (NF-κB) signaling plays a vital role in both immunity and inflammation (67). Upon stimulation, NF-κB translocates into the nucleus and promotes the transcription of numerous genes critical for dendritic cell function, T cell activation and sustainment of secondary lymphoid organs. In a study performed by Wang et al., TNF-α induced phosphorylation of IκBα and production of P65 in Hep3 B cells. Artemisinin suppressed the activation of NF-κB pathway induced by TNF-α *via* attenuating IκBα phosphorylation and activation of P65, resulting in a decrease in expression of NF-κB target genes and two adaptor proteins, RAF2 and RIP1, which in turn exerted their effects on upstream of IKK signaling (68). Dihydroartemisinin and artesunate also inhibited NF-*κ*B signaling, leading to alleviation of colitis. Dihydroartemisinin significantly inhibited phosphorylation of IKK*α*, I*κ*B*α*, and NF-*κ*B (p65) in DSS-induced murine colitis and IEC-6 cells treated with LPS (37) while artesunate remarkably suppressed the NF-κB activation with a reduction in mRNA expression of IL-1β, IL-6 and TNF-α and an increase in IL-10 gene expression (41, 44, 45). Taken together, ARTs can hinder activation of NF-κB pathway and therefore suppress inflammatory responses *in vivo* and *in vitro* (**Figure 2**).

### Nrf2

Transcription factor Nrf2, which is negatively regulated by Keap1, plays an important role in controlling the expression of antioxidant enzymes and suppressing inflammation *via* regulating transcription of anti-inflammatory genes (69, 70) while P62-mediated Keap1 degradation contributes to nuclear translocation of Nrf2 and related gene transcriptions. Dihydroartemisinin treatment elevated the expression of Nrf2 and its target gene HO-1 in MDSCs from SLE mice and attenuated the senescence of MDSCs (54). It was also found that artesunate activated Nrf2 by augmenting p62 expression in murine bone marrow macrophages, resulting in an increase in the expression of HO-1 or NQO1 (19). Similarly, DC32 strengthened Nrf2/HO-1 signaling and promoted p62 transcription or Keap1 degradation in DBA/1 mice as well as NIH-3T3 cells (21). Collectively, ARTs promote the activation of Nrf2 signaling, resulting in elevated expression of anti-inflammatory genes and a reduction in inflammation (**Figure 2**).

### JAK/STAT

Janus kinase (JAK) and signal transducer and activator of transcription (STAT) proteins control signal transduction of many cytokines and growth factors associated with cellular growth, survival and differentiation (71). Artesunate reportedly attenuated STAT1 phosphorylation in cultured HUVECs stimulated with IFNα (56) and suppressed phosphorylation of JAK2 and STAT3 in the kidney of MRL/lpr mice, resulting in an amelioration of the symptoms of lupus nephritis (57).

### mTOR

Mammalian or mechanistic target of rapamycin (mTOR) is a protein kinase that regulates eukaryotic signaling networks and diverse cellular processes upon environmental changes (72). Previous studies demonstrated that artesunate not only downregulated the mRNA and protein expressions of PI3K, AKT and mTOR, but also inhibited phosphorylation of these proteins in the cartilage tissue of RA mice and chondrocytes *in vitro* (18), while dihydroartemisinin suppressed phosphorylation of mTOR and S6K in human mesangial cells in the presence of IgA1 stimulation (63). Thus, ARTs may serve as a novel inhibitor of mTOR.

## Conclusions and Perspectives

The number of patients treated with immunosuppressive drugs for immune-mediated inflammatory diseases (IMIDs) and transplant rejection has gradually increased over the last decades (73). IMIDs or autoimmune diseases affect 5-7% of the population in western countries (12), resulting in substantial personal and societal costs due to disease chronicity. Lifelong administration of conventional immunosuppressive agents poses a huge economic burden to patients with autoimmune diseases or organ transplantation, accompanied by some complications, such as infections, tumors and lymphoproliferative diseases. In spite of advances in remedies, IMIDs and allograft rejection remain linked to a high risk of morbidity and mortality (74). Thus, finding a cost-effective and efficacious treatment with few side effects is warranted.

ARTs have been widely used in humans for treating malaria with only mild side effects (75). Other clinical trials showed that artesunate was safe and well-tolerated in patients with breast cancer (76, 77), while artemisinin treatment in pregnant women did not elevate the risk of miscarriage, stillbirth or congenital malformation (78). Collectively, ARTs are generally considered to be safe and effective in clinical practice.

In this review, we summarize the latest studies demonstrating the efficacy of ARTs in the treatment of IMIDs or autoimmune diseases and allograft rejection. ARTs mainly regulate adaptive and innate immune cells, including subsets of CD4+ T cells (Th1/Th2/Th9/Th17/Tfh/Tfr/Treg), CD8+ memory T cells, γδ T cells, B cells, dendritic cells, neutrophils, mast cells, macrophages, and MDSCs, through altering cellular apoptosis and differentiation, pro-inflammatory and anti-inflammatory cytokine secretion, and signal transduction. So far, systematic researches into ARTs for their efficacy in IMIDs and allograft rejection have been largely confined to animal models due to the lack of large randomized and controlled clinical trials, although previous clinical evidence has indicated that ARTs may have been immunosuppressive in lupus patients (79–81). Therefore, more clinical studies are warranted to evaluate their doses, efficacy and side effects in the treatment of IMIDs and allograft rejection. ARTs may present a promising therapeutic alternative for treating IMIDs and allograft rejection in the near future. Alternatively, they could be used in combination with an immunosuppressant to either enhance therapeutic efficacy or reduce side effects.

## Author Contributions

FQ and JL wrote the original manuscript together. XM, HL, and YC searched some of the literature. ZD provided the general idea and edited the manuscript. All authors contributed to the article and approved the submitted version.

## Funding

This work was supported by National Natural Science Foundation of China (82071800), the State Key Laboratory of Dampness Syndrome of Chinese Medicine (SZ2020ZZ16 and SZ2020ZZ18), the 2020 Guangdong Provincial Science and Technology Innovation Strategy Special Fund (Guangdong-Hong Kong-Macau Joint Lab 2020B1212030006), the Specific Research Fund for TCM Science and Technology of Guangdong Provincial Hospital of Chinese Medicine (YN2019MJ03, YN2019QJ07 and YN2019QJ02), and the Key-Area Research and Development Program of Guangdong Province (NO. 2020B1111100010). 

## Conflict of Interest

The authors declare that the research was conducted in the absence of any commercial or financial relationships that could be construed as a potential conflict of interest.

## Publisher’s Note

All claims expressed in this article are solely those of the authors and do not necessarily represent those of their affiliated organizations, or those of the publisher, the editors and the reviewers. Any product that may be evaluated in this article, or claim that may be made by its manufacturer, is not guaranteed or endorsed by the publisher.
